# Supplementary material for: Novel Design of an α-Amylase with an N-Terminal CBM20 in Aspergillus niger Improves Binding and Processing of a Broad Range of Starches
Source: Molecules. 2023 Jun 27;28(13):5033. doi: 10.3390/molecules28135033 (PMC10343422; doi:10.3390/molecules28135033)
Supplement: Supplementary file 1 [file molecules-28-05033-s001.zip › molecules-2341845-supplementary.pdf]

Table S1. Purification scheme of GH13 α-amylase variants based on the activity resulted in purification steps. The α-amylase activity was measured toward (A.1-4.) 2-Chloro-4-nitrophenyl-β-D-maltotrioside (CNPG3); (B.1-4.) Soluble starch; (C.1-4.) Rice starch; (D.1-4.) Corn starch; (E.1-4.) Wheat starch; (F.1-4.) Potato starch.

(A)[a]

**(A.1) CBM20_GH13**	Volume (mL)	Nitrophenol release (mM)	Amylase activity (U/mL,hr)	Total activity (U)	Protein (mg/mL)	Total protein (mg)	Specific activity (U/mg,hr)	Enzyme recovery (%)	
Spent medium	300	0.8	15.1	4534	0.17	51.1	89	100	
Unbound fraction	300	0.3	6.1	1834	0.15	45.8	40	40	*Unbound protein:40%
Washed fraction	300	0.003	0.1	17	0.006	1.9	8.5	0	*Bound protein: 60%
Eluted protein	15	4.8	96.2	1443	0.02	0.3	4815	32	
Rest activity (likely remained in starch)				1241				27	

**(A.2) GH13_CBM20**	Volume (mL)	Nitrophenol release (mM)	Amylase activity (U/mL,hr)	Total activity (U)	Protein (mg/mL)	Total Protein (mg)	Specific activity (U/mg,hr)	Enzyme recovery (%)	
Spent medium	300	0.9	18.4	5513	0.21	64.5	85	100	
Unbound fraction	300	0.5	10.1	3022	0.18	52.7	57	55	*Unbound protein: 56%
Washed fraction	300	0.01	0.2	48	0.01	2.6	18	1	*Bound protein: 44%
Eluted protein	15	5.7	113.1	1697	0.03	0.44	3886	31	
Rest activity (likely remained in starch)				746				14	

**(A.3) GH13**	Volume (mL)	Nitrophenol release (mM)	Amylase activity (U/mL,hr)	Total activity (U)	Protein (mg/mL)	Total Protein (mg)	Specific activity (U/mg,hr)	Enzyme recovery (%)	
Spent medium	300	0.3	6.1	1839	0.17	52.5	35	100	
Unbound	300	0.3	5.6	1684	0.17	51.3	33	92	*Unbound protein: 92%
Washed	300	0.001	0.012	3	0.01	2.0	2	0	*Bound protein: 8%
Eluted protein	15	0.4	8.3	124	0.005	0.1	1732	7	
Rest Activity (likely remained in starch)				28				2	

**(A.4) MGG029** (parental strain with empty vector)	Volume (mL)	Nitrophenol release (mM)	Amylase activity (U/mL,hr)	Total activity (U)	Protein (mg/mL)	Total Protein (mg)	Specific activity (U/mg,hr)	Enzyme recovery (%)	
Spent medium	300	0.1	2.8	827.6	0.16	49.5	17	100	
Unbound	300	0.1	2.7	809.4	0.16	49.4	16	97.8	*Unbound protein: 98%
Washed	300	0.0	0.0	0.08	0.00	1.0	0.0	0.01	*Bound protein: 2%
Eluted protein	15	0.1	1.2	17.8	0.003	0.04	423	2.15	
Rest Activity (likely remained in starch)				0.3				0.04	

*Unbound protein (%) is the total of unbound fraction and washed protein; *Bound protein: [Total activity of spent medium (in 100%) – total activity of unbound (%)].

[a]These activities were measured based on the corn-starch binding purification

(B)[a]

(B.1) CBM20_GH13	Volume (mL)	Reducing sugar release (mM)	Amylase activity (U/mL,hr)	Total activity (U)	Protein (mg/mL)	Total Protein (mg)	Specific activity (U/mg,hr)	Enzyme recovery (%)	
Spent medium	300	0.2	3.3	997.4	0.17	51.1	19.5	100	
Unbound	300	0.1	1.4	420.4	0.15	45.8	9.2	42	*Unbound protein: 42%
Washed	300	0.0	0.0	0.2	0.006	1.9	0.1	0.0	*Bound protein: 58%
Eluted protein	15	1.0	21.0	314.7	0.02	0.3	1125.6	32	
Rest Activity (likely remained in starch)				262.1				26	

(B.2) GH13_CBM20	Volume (mL)	Reducing sugar release (mM)	Amylase activity (U/mL,hr)	Total activity (U)	Protein (mg/mL)	Total Protein (mg)	Specific activity (U/mg,hr)	Enzyme recovery (%)	
Spent medium	300	0.2	4.3	1278.0	0.22	67.4	19.0	100	
Unbound	300	0.1	2.3	693.7	0.18	53.0	13.1	54	*Unbound protein: 56%
Washed	300	0.0	0.1	27.5	0.01	2.6	10.5	2.2	*Bound protein: 44%
Eluted protein	15	1.2	23.6	353.3	0.03	0.4	902.0	28	
Rest Activity (likely remained in starch)				203.6				16	

(B.3) GH13	Volume (mL)	Reducing sugar release (mM)	Amylase activity (U/mL,hr)	Total activity (U)	Protein (mg/mL)	Total Protein (mg)	Specific activity (U/mg,hr)	Enzyme recovery (%)	
Spent medium	300	0.1	1.6	486.3	0.17	52	9.3	100	
Unbound	300	0.1	1.5	444.9	0.17	51	8.7	91	*Unbound protein: 92%
Washed	300	0.0	0.0	4.5	0.01	2	2.3	0.9	*Bound protein: 8%
Eluted protein	15	0.1	2.4	35.6	0.005	0.1	432.5	7	
Rest Activity (likely remained in starch)				1.3				0	

**(B.4) MGG029** (parental strain with empty vector)	Volume (mL)	Reducing sugar release (mM)	Amylase activity (U/mL,hr)	Total activity (U)	Protein (mg/mL)	Total Protein (mg)	Specific activity (U/mg,hr)	Enzyme recovery (%)	
Spent medium	300	0.0	0.7	203.6	0.16	49	4.1	100.0	
Unbound	300	0.0	0.7	197.9	0.16	49	4.0	97.2	*Unbound protein: 97%
Washed	300	0.0	0.0	0.0	0.00	1	0.0	0.0	*Bound protein: 3%
Eluted protein	15	0.0	0.4	5.4	0.003	0.0	133.1	2.7	
Rest Activity (likely remained in starch)				0.3				0.1	

*Unbound protein (%) is the total of unbound fraction and washed protein; *Bound protein: [Total activity of spent medium (in 100%) – total activity of unbound (%)]

[a]These activities were measured based on the corn-starch binding purification

(C)[a]

**(C.1) CBM20_GH13**	Volume (mL)	Reducing sugar release (mM)	Amylase activity (U/mL,hr)	Total activity (U)	Protein (mg/mL)	Total Protein (mg)	Specific activity (U/mg,hr)	Enzyme recovery (%)	
Spent medium	300	0.3	5.6	1670.1	0.17	51.1	32.7	100	
Unbound	300	0.1	2.5	748.4	0.15	45.8	16.3	45	*Unbound protein: 45%
Washed	300	0.0	0.0	7.5	0.006	1.9	3.9	0	*Bound protein: 55%
Eluted protein	15	1.63	32.6	489.7	0.02	0.28	1751.6	29	
Rest Activity (likely remained in starch)				424.5				25	

**(C.2) GH13_CBM20**	Volume (mL)	Reducing sugar release (mM)	Amylase activity (U/mL,hr)	Total activity (U)	Protein (mg/mL)	Total Protein (mg)	Specific activity (U/mg,hr)	Enzyme recovery (%)	
Spent medium	300	0.4	7.0	2113.1	0.22	67.4	31.4	100	
Unbound	300	0.2	4.1	1219.6	0.18	53.0	23.0	58	*Unbound protein: 60%
Washed	300	0.0	0.2	56.6	0.01	2.6	21.6	2.7	*Bound protein: 40%
Eluted protein	15	1.9	37.2	558.5	0.03	0.4	1426.1	26	
Rest Activity (likely remained in starch)				278.4				13	

**(C.3) GH13**	Volume (mL)	Reducing sugar release (mM)	Amylase activity (U/mL,hr)	Total activity (U)	Protein (mg/mL)	Total Protein (mg)	Specific activity (U/mg,hr)	Enzyme recovery (%)	
Spent medium	300	0.2	4.2	1264.8	0.17	52	24.1	100	
Unbound	300	0.2	3.9	1165.7	0.17	51	22.7	92	*Unbound protein: 92%
Washed	300	0.0	0.0	3.2	0.01	2	1.6	0.3	*Bound protein: 8%
Eluted protein	15	0.3	6.0	90.7	0.005	0.1	1217.6	7	
Rest Activity (likely remained in starch)				5.3				0	

**(C.4) MGG029** (parental strain with empty vector)	Volume (mL)	Reducing sugar release (mM)	Amylase activity (U/mL,hr)	Total activity (U)	Protein (mg/mL)	Total Protein (mg)	Specific activity (U/mg,hr)	Enzyme recovery (%)	
Spent medium	300	0.1	2.7	808.7	0.16	49	16.3	100.0	
Unbound	300	0.1	2.6	793.6	0.16	49	16.1	98.1	*Unbound protein: 98%
Washed	300	0.0	0.0	0.0	0.00	1	0.0	0.0	*Bound protein: 2%
Eluted protein	15	0.0	1.0	14.5	0.003	0.0	318.3	1.8	
Rest Activity (likely remained in starch)				0.6				0.1	

*Unbound protein (%) is the total of unbound fraction and washed protein; *Bound protein: [Total activity of spent medium (in 100%) – total activity of unbound (%)]

[a]These activities were measured based on the corn-starch binding purification

(D)[a]

**(D.1) CBM20_GH13**	Volume (mL)	Reducing sugar release (mM)	Amylase activity (U/mL,hr)	Total activity (U)	Protein (mg/mL)	Total Protein (mg)	Specific activity (U/mg,hr)	Enzyme recovery (%)	
Spent medium	300	0.1	2.0	613.2	0.17	51.1	12.0	100	
Unbound	300	0.0	0.8	248.8	0.15	45.8	5.4	41	*Unbound protein: 41%
Washed	300	0.0	0.0	2.5	0.006	1.9	1.3	0	*Bound protein: 59%
Eluted protein	15	0.6	12.7	191.2	0.02	0.3	684.0	31	
Rest activity (likely remained in starch)				170.7				28	

**(D.2) GH13_CBM20**	Volume (mL)	Reducing sugar release (mM)	Amylase activity (U/mL,hr)	Total activity (U)	Protein (mg/mL)	Total Protein (mg)	Specific activity (U/mg,hr)	Enzyme recovery (%)	
Spent medium	300	0.1	2.2	663.5	0.22	67.4	9.8	100	
Unbound	300	0.1	1.3	384.5	0.18	53.0	7.3	58	*Unbound protein: 58%
Washed	300	0.0	0.0	0.0	0.01	2.6	0.0	0.0	*Bound protein: 42%
Eluted protein	15	0.7	14.0	210.6	0.03	0.4	537.8	32	
Rest activity (likely remained in starch)				68.3				10	

**(D.3) GH13**	Volume (mL)	Reducing sugar release (mM)	Amylase activity (U/mL,hr)	Total activity (U)	Protein (mg/mL)	Total Protein (mg)	Specific activity (U/mg,hr)	Enzyme recovery (%)	
Spent medium	300	0.0	0.8	248.8	0.17	52	4.7	100.0	
Unbound	300	0.0	0.8	231.9	0.17	51	4.5	93.2	*Unbound protein: 93%
Washed	300	0.0	0.0	0.0	0.01	2	0.0	0.0	*Bound protein: 7%
Eluted protein	15	0.1	1.0	15.7	0.005	0.1	207.1	6.3	
Rest activity (likely remained in starch)				1.2				0.5	

**(D.4) MGG029** (parental strain with empty vector)	Volume (mL)	Reducing sugar release (mM)	Amylase activity (U/mL,hr)	Total activity (U)	Protein (mg/mL)	Total Protein (mg)	Specific activity (U/mg,hr)	Enzyme recovery (%)	
Spent medium	300	0.0	0.4	105.6	0.16	49	2.1	100.0	
Unbound	300	0.0	0.3	101.8	0.16	49	2.1	96.4	*Unbound protein: 96%
Washed	300	0.0	0.0	0.0	0.00	1	0.0	0.0	*Bound protein: 4%
Eluted protein	15	0.0	0.2	3.7	0.003	0.1	71.6	3.5	
Rest activity (likely remained in starch)				0.1				0.1	

*Unbound protein (%) is the total of unbound fraction and washed protein; *Bound protein: [Total activity of spent medium (in 100%) – total activity of unbound (%)]

[a]These activities were measured based on the corn-starch binding purification

(E)[a]

**(E.1) CBM20_GH13**	Volume (mL)	Reducing sugar release (mM)	Amylase activity (U/mL,hr)	Total activity (U)	Protein (mg/mL)	Total Protein (mg)	Specific activity (U/mg,hr)	Enzyme recovery (%)	
Spent medium	300	0.1	1.2	348.7	0.17	51.1	6.8	100	
Unbound	300	0.0	0.5	156.6	0.15	45.8	3.4	45	*Unbound protein: 45%
Washed	300	0.0	0.0	0.0	0.006	1.9	0.0	0	*Bound protein: 55%
Eluted protein	15	0.3	6.9	103.6	0.02	0.3	370.4	30	
Rest Activity (likely remained in starch)				88.5				25	

**(E.2) GH13_CBM20**	Volume (mL)	Reducing sugar release (mM)	Amylase activity (U/mL,hr)	Total activity (U)	Protein (mg/mL)	Total Protein (mg)	Specific activity (U/mg,hr)	Enzyme recovery (%)	
Spent medium	300	0.1	1.3	394.0	0.22	67.4	5.8	100	
Unbound	300	0.0	0.7	218.7	0.18	53.0	4.1	56	*Unbound protein: 57%
Washed	300	0.0	0.0	3.8	0.01	2.6	1.4	1.0	*Bound protein: 43%
Eluted protein	15	0.4	7.2	107.8	0.03	0.4	275.3	27	
Rest Activity (likely remained in starch)				63.7				16	

**(E.3) GH13**	Volume (mL)	Reducing sugar release (mM)	Amylase activity (U/mL,hr)	Total activity (U)	Protein (mg/mL)	Total Protein (mg)	Specific activity (U/mg,hr)	Enzyme recovery (%)	
Spent medium	300	0.0	0.4	132.0	0.17	52.5	2.5	100.0	
Unbound	300	0.0	0.4	122.5	0.17	51.3	2.4	92.9	*Unbound protein: 93%
Washed	300	0.0	0.0	0.0	0.01	2	0.0	0.0	*Bound protein: 7%
Eluted protein	15	0.0	0.6	9.2	0.005	0.1	129.2	7.0	
Rest Activity (likely remained in starch)				0.2				0.1	

**(E.4) MGG029** (parental strain with empty vector)	Volume (mL)	Reducing sugar release (mM)	Amylase activity (U/mL,hr)	Total activity (U)	Protein (mg/mL)	Total Protein (mg)	Specific activity (U/mg,hr)	Enzyme recovery (%)	
Spent medium	300	0.0	0.2	71.6	0.16	49	1.4	100	
Unbound	300	0.0	0.2	68.8	0.16	49	1.4	96	*Unbound protein: 96%
Washed	300	0.0	0.0	0.2	0.00	1	0.2	0.3	*Bound protein: 4%
Eluted protein	15	0.0	0.2	2.5	0.003	0.0	54.1	4	
Rest Activity (likely remained in starch)				0				0	

*Unbound protein (%) is the total of unbound fraction and washed protein; *Bound protein : [Total activity of spent medium (in 100%) – total activity of unbound (%)]

[a]These activities were measured based on the corn-starch binding purification

(F)[a]

**(F.1) CBM20_GH13**	Volume (mL)	Reducing sugar release (mM)	Amylase activity (U/mL,hr)	Total activity (U)	Protein (mg/mL)	Total Protein (mg)	Specific activity (U/mg,hr)	Enzyme recovery (%)	
Spent medium	300	0.0	0.6	167.8	0.17	51.1	3.3	100	
Unbound	300	0.0	0.3	77.3	0.15	45.8	1.7	46	*Unbound protein: 46%
Washed	300	0.0	0.0	0.02	0.006	1.9	0.0	0	*Bound protein: 54%
Eluted protein	15	0.16	3.3	49.5	0.02	0.3	177.0	29	
Rest Activity (likely remained in starch)				41.0				24	

**(F.2) GH13_CBM20**	Volume (mL)	Reducing sugar release (mM)	Amylase activity (U/mL,hr)	Total activity (U)	Protein (mg/mL)	Total Protein (mg)	Specific activity (U/mg,hr)	Enzyme recovery (%)	
Spent medium	300	0.0	0.4	126.3	0.22	67.4	1.9	100	
Unbound	300	0.0	0.2	71.6	0.18	53.0	1.4	57	*Unbound protein: 57%
Washed	300	0.0	0.0	0.1	0.01	2.6	0.0	0.07	*Bound protein: 43%
Eluted protein	15	0.1	2.2	32.7	0.03	0.4	83.5	26	
Rest Activity (likely remained in starch)				21.9				17	

**(F.3) GH13**	Volume (mL)	Reducing sugar release (mM)	Amylase activity (U/mL,hr)	Total activity (U)	Protein (mg/mL)	Total Protein (mg)	Specific activity (U/mg,hr)	Enzyme recovery (%)	
Spent medium	300	0.0	0.2	56.6	0.17	52	1.1	100	
Unbound	300	0.0	0.2	54.7	0.17	51	1.1	97	*Unbound protein: 97%
Washed	300	0.0	0.0	0.0	0.01	2	0.0	0.0	*Bound protein: 3%
Eluted protein	15	0.0	0.1	1.8	0.005	0.1	25.7	3	
Rest Activity (likely remained in starch)				0.1				0	

**(F.4) MGG029** (parental strain with empty vector)	Volume (mL)	Reducing sugar release (mM)	Amylase activity (U/mL,hr)	Total activity (U)	Protein (mg/mL)	Total Protein (mg)	Specific activity (U/mg,hr)	Enzyme recovery (%)	
Spent medium	300	0.0	0.1	29.2	0.16	49	0.6	100.0	
Unbound	300	0.0	0.1	28.8	0.16	49	0.6	98.5	*Unbound protein: 98%
Washed	300	0.0	0.0	0.00	0.00	1	0.0	0.0	*Bound protein: 2%
Eluted protein	15	0.0	0.0	0.4	0.003	0.1	8.0	1.4	
Rest Activity (likely remained in starch)				0.0				0.0	

*Unbound protein (%) is the total of unbound fraction and washed protein; *Bound protein : [Total activity of spent medium (in 100%) – total activity of unbound (%)]

[a]These activities were measured based on the corn-starch binding purification

Figure S1. Amino acid sequence of α-amylase variants used in this study

>Chimeric CBM20_GH13 (New design)
MSFRSLLALSGLVCTGLATVLHRHGHQGRHIHARQDCATVAVTFDLTATTTYGENIYLVGSISQLGDWETSDGIAL
SADKYTSSDPLWYVTVTLPAGESFEYKFIRIESDDSVEWESDPNREYTVPQACGTSTATVTDTWVSQEQWWCSEDD
PAAVAASQAARVYMDCHPKPRHPRKPIPVFVPDWRTQSIYFLLTDRFGRTDNSTTATCDTGDQIYCGGSWQGIINH
LDYIQGMGFTAIWISPITEQLPQDTADGEAYHGYWQQKIYDVNSNFGTADDLKSLSDALHARGMYLMVDVVPNHMG
YAGNGNDVDYSVFDPFDSSSYFHPYCLITDWDNLTMVQDCWEGDTIVSLPDLNTTETAVRTIWYDWVADLVSNYSV
DGLRIDSVLEVEPDFFPGYQEAAGVYCVGEVDNGNPALDCPYQKVLDGVLNYPIYWQLLYAFESSSGSISNLYNMI
KSVASDCSDPTLLGNFIENHDNPRFASYTSDYSQAKNVLSYIFLSDGIPIVYAGEEQHYSGGKVPYNREATWLSGY
DTSAELYTWIATTNAIRKLAISADSAYITYANDAFYTDSNTIAMRKGTSGSQVITVLSNKGSSGSSYTLTLSGSGY
TSGTKLIEAYTCTSVTVDSSGDIPVPMASGLPRVLLPASVVDSSSLCGGS

>GH13 (Native_ ANI_1_460094)
MRLSTSSLFLSVSLLGKLALGLSAAEWRTQSIYFLLTDRFGRTDNSTTATCDTGDQIYCGGSWQGIINHLDYIQGM
GFTAIWISPITEQLPQDTADGEAYHGYWQQKIYDVNSNFGTADDLKSLSDALHARGMYLMVDVVPNHMGYAGNGND
VDYSVFDPFDSSSYFHPYCLITDWDNLTMVQDCWEGDTIVSLPDLNTTETAVRTIWYDWVADLVSNYSVDGLRIDS
VLEVEPDFFPGYQEAAGVYCVGEVDNGNPALDCPYQKVLDGVLNYPIYWQLLYAFESSSGSISNLYNMIKSVASDC
SDPTLLGNFIENHDNPRFASYTSDYSQAKNVLSYIFLSDGIPIVYAGEEQHYSGGKVPYNREATWLSGYDTSAELY
TWIATTNAIRKLAISADSAYITYANDAFYTDSNTIAMRKGTSGSQVITVLSNKGSSGSSYTLTLSGSGYTSGTKLI
EAYTCTSVTVDSSGDIPVPMASGLPRVLLPASVVDSSSLCGGS

>GH13_CBM20 (Native, as reference_ KAI3001921.1)
MRLSTSSLLLSVSLLGKLALGLSAAEWRTQSIYFLLTDRFGRTDNSTTATCNTGDQIYCGGSWQGIINHLDYIQGM
GFTAIWISPITEQLPQDTADGEAYHGYWQQKIYDVNSNFGTADDLKSLSDALHARGMYLMVDVVPNHMGYAGNGND
VDYSVFDPFDSSSYFHPYCLITDWDNLTMVQDCWEGDTIVSLPDLNTTETAVRTIWYDWVADLVSNYSVDGLRIDS
VLEVEPDFFPGYQEAAGVYCVGEVDNGNPALDCPYQEYLDGVLNYPIYWQLLYAFESSSGSISDLYNMIKSVASDC
SDPTLLGNFIENHDNPRFASYTSDYSQAKNVLSYIFLSDGIPIVYAGEEQHYSGGKVPYNREATWLSGYDTSAELY
TWIATTNAIRKLAISADSAYITYANDAFYTDSNTIAMRKGTSGSQVITVLSNKGSSGSSYTLTLSGSGYTSGTKLI
EAYTCTSVTVDSSGDIPVPMASGLPRVLLPASVVDSSSLCGGSGSNSSTTTTTTATSSSTATSKSASTSSTSTACT
ATSTSLAVTFEELVTTTYGEEIYLSGSISQLGDWDTSDAVKMSADDYTSSNPEWSVTVTLPVGTTFEYKFIKVESD
GTVTWESDPNREYTVPECGSGETVVDTWR

Yellow: Signal sequence

Grey: linker

Purple: CBM20 chimeric and native

Green: GH13

Figure S2. Multiple alignment of protein sequence**. (a).** Multiple sequence alignment of modular GH13 α-amylase variants from the new and native design. Both N- and C-terminal CBM20 domain are marked with purple font. GH13 α-amylase domain are marked with green font. Sequence identity is indicated by asterisks, while sequence similarity is marked with a dot (PDB code 2AAA). **(b).** Sequence alignment of CBM20 domains from the new CBM20_GH13 and native GH13_CBM20 design. Sequence identities are indicated by asterisks and black font. Non-identical sequences are marked with dot and red font. The amino acid residues involved in substrate binding are highlighted in soft blue and all are conserved for both N and C-terminus CBM20 used in this study. These four essential binding sites were predicted based on the crystal structure of CBM20 from A. niger glucoamylase that was also applied for the CBM20_GH13 design (PDB code 1AC0)

(a.)

```
CBM20_GH13 (new)      QDCATVAVTFDLTATTTYGENIYLVGSISQLGDWETSDGIALSADKYTSSDPLWYVTVTL

GH13_CBM20 (native)   ------------------------------------------------------------
GH13 (native)         ------------------------------------------------------------

CBM20_GH13 (new)      PAGESFEYKFIRIESDDSVEWESDPNREYTVPQACGTSTATVTDTWVSQEQWWCSEDDPA
GH13_CBM20 (native)   ------------------------------------------------------------
GH13 (native)         ------------------------------------------------------------

CBM20_GH13 (new)      AVAASQAARVYMDCHPKPRHPRKPIPVFVPDWRTQSIYFLLTDRFGRTDNSTTATCDTGD
GH13_CBM20 (native)   -------------------------------WRTQSIYFLLTDRFGRTDNSTTATCNTGD
GH13 (native)         -------------------------------WRTQSIYFLLTDRFGRTDNSTTATCDTGD
                                                     *************************.***

CBM20_GH13 (new)      QIYCGGSWQGIINHLDYIQGMGFTAIWISPITEQLPQDTADGEAYHGYWQQKIYDVNSNF
GH13_CBM20 (native)  QIYCGGSWQGIINHLDYIQGMGFTAIWISPITEQLPQDTADGEAYHGYWQQKIYDVNSNF
GH13 (native)         QIYCGGSWQGIINHLDYIQGMGFTAIWISPITEQLPQDTADGEAYHGYWQQKIYDVNSNF
                      ************************************************************

CBM20_GH13 (new)      GTADDLKSLSDALHARGMYLMVDVVPNHMGYAGNGNDVDYSVFDPFDSSSYFHPYCLITD
GH13_CBM20 (native)   GTADDLKSLSDALHARGMYLMVDVVPNHMGYAGNGNDVDYSVFDPFDSSSYFHPYCLITD
GH13 (native)         GTADDLKSLSDALHARGMYLMVDVVPNHMGYAGNGNDVDYSVFDPFDSSSYFHPYCLITD
                      ************************************************************

CBM20_GH13 (new)      WDNLTMVQDCWEGDTIVSLPDLNTTETAVRTIWYDWVADLVSNYSVDGLRIDSVLEVEPD
GH13_CBM20 (native)   WDNLTMVQDCWEGDTIVSLPDLNTTETAVRTIWYDWVADLVSNYSVDGLRIDSVLEVEPD
GH13 (native)         WDNLTMVQDCWEGDTIVSLPDLNTTETAVRTIWYDWVADLVSNYSVDGLRIDSVLEVEPD
                      ************************************************************

CBM20_GH13 (new)      FFPGYQEAAGVYCVGEVDNGNPALDCPYQKVLDGVLNYPIYWQLLYAFESSSGSISNLYN
GH13_CBM20 (native)   FFPGYQEAAGVYCVGEVDNGNPALDCPYQEYLDGVLNYPIYWQLLYAFESSSGSISDLYN
GH13 (native)         FFPGYQEAAGVYCVGEVDNGNPALDCPYQKVLDGVLNYPIYWQLLYAFESSSGSISNLYN
                      *****************************..*************************.***

CBM20_GH13 (new)      MIKSVASDCSDPTLLGNFIENHDNPRFASYTSDYSQAKNVLSYIFLSDGIPIVYAGEEQH
GH13_CBM20 (native)   MIKSVASDCSDPTLLGNFIENHDNPRFASYTSDYSQAKNVLSYIFLSDGIPIVYAGEEQH
GH13 (native)         MIKSVASDCSDPTLLGNFIENHDNPRFASYTSDYSQAKNVLSYIFLSDGIPIVYAGEEQH
                      ************************************************************

CBM20_GH13 (new)      YSGGKVPYNREATWLSGYDTSAELYTWIATTNAIRKLAISADSAYITYANDAFYTDSNTI
GH13_CBM20 (native)  YSGGKVPYNREATWLSGYDTSAELYTWIATTNAIRKLAISADSAYITYANDAFYTDSNTI
GH13 (native)         YSGGKVPYNREATWLSGYDTSAELYTWIATTNAIRKLAISADSAYITYANDAFYTDSNTI
                      ************************************************************
```

```
CBM20_GH13 (new)     AMRKGTSGSQVITVLSNKGSSGSSYTLTLSGSGYTSGTKLIEAYTCTSVTVDSSGDIPVP
GH13_CBM20 (native)  AMRKGTSGSQVITVLSNKGSSGSSYTLTLSGSGYTSGTKLIEAYTCTSVTVDSSGDIPVP
GH13 (native)        AMRKGTSGSQVITVLSNKGSSGSSYTLTLSGSGYTSGTKLIEAYTCTSVTVDSSGDIPVP
                     ************************************************************

CBM20_GH13 (new)     MASGLPRVLLPASVVDSSSLCGGS------------------------------------
GH13_CBM20 (native)  MASGLPRVLLPASVVDSSSLCGGSGSNSSTTTTTATSSSTATSKSASTSSTSTACTATS
GH13 (native)        MASGLPRVLLPASVVDSSSLCGGS------------------------------------
                     ************************

CBM20_GH13 (new)     ------------------------------------------------------------
GH13_CBM20 (native)  TSLAVTFEELVTTTYGEEIYLSGSISQLGDWDTSDAVKMSADDYTSSNPEWSVTVTLPVG
GH13 (native)        ------------------------------------------------------------

CBM20_GH13 (new)     ------------------------------------------
GH13_CBM20 (native)  TTFEYKFIKVESDGTVTWESDPNREYTVPECGSGETVVDTWR
GH13 (native)        ------------------------------------------
```

(b)

```
CBM20 of New CBM20_GH13       AVTFDLTATTTYGENIYLVGSISQLGDWETSDGIALSADKYTSSDPLWYVTVTL
CBM20 of Native GH13_CBM20    AVTFEELVTTTYGEEIYLSGSISQLGDWDTSDAVKMSADDYTSSNPEWSVTVTL
                              ****....******.***.*********.***....***.****.*.*.*****

CBM20 of New CBM20_GH13       PAGESFEYKFIRIESDDSVEWESDPNREYTVPQACGTSTATVTDTW
CBM20 of Native GH13_CBM20    PVGTTFEYKFIKVESDGTVTWESDPNREYTVPE-CG-SGETVVDTW
                              *.*..******..***..*.************. ** *..**.***
```

Figure S3. SDS-PAGE of purified α-amylase variants obtained by starch binding purification. Gels were stained with Sypro Ruby and the α-amylase bands were identified because of the absence of bands in these positions in the parental strain transformed with the empty vector. Lane 1 is molecular weight markers; lane 2 is CBM20_GH13, lane3 is GH13_CBM20, lane 4 is GH13, and Lane 5 is the parental strain *A. niger* MGG029 with empty vector as a background/ - control

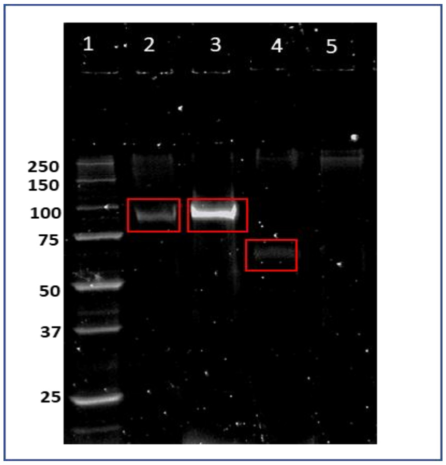

Figure S4. SDS-PAGE of 0.5 ug purified α-amylase obtained by starch binding purification. Gels were stained with Sypro Ruby and the α-amylase bands were identified because of the absence of bands in these positions in the parental strain transformed with the empty vector. Lane 1: molecular weight marker, Lane2: CBM20_GH13, Lane3: GH13_CBM20.

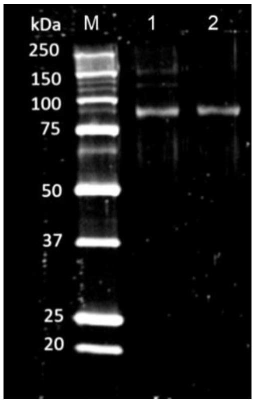

Figure S5. Kinetic Rate of Enzyme on Various Substrates. (**A**) 2-Chloro-4-nitrophenyl-β-D-maltotrioside (CNPG3), (**B**) Soluble Starch, (**C**) Rice starch, (**D**) Corn starch, (**E**) Wheat starch, (**F**) Potato starch.

(A)

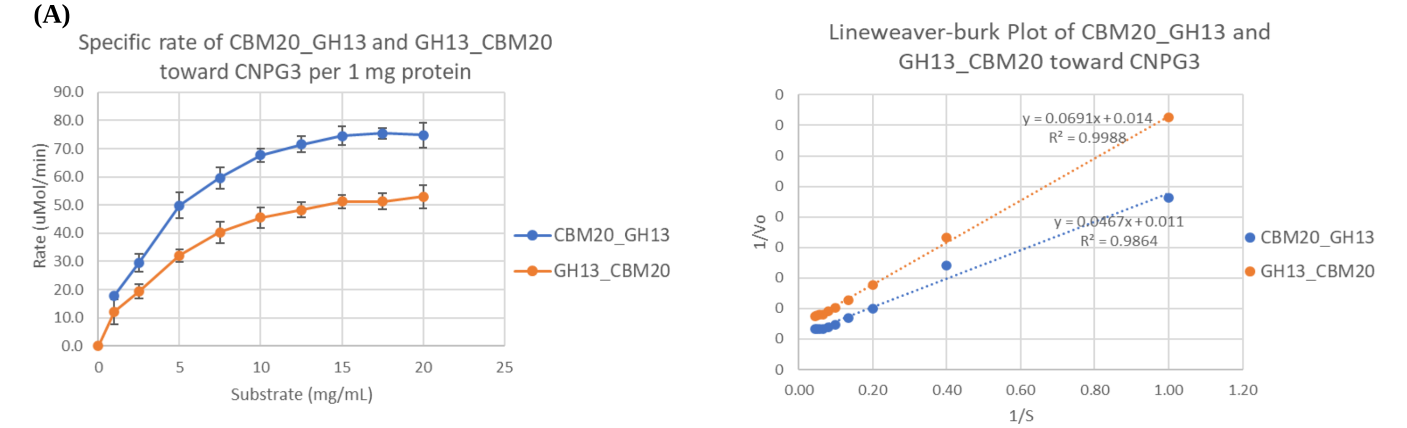

(B)

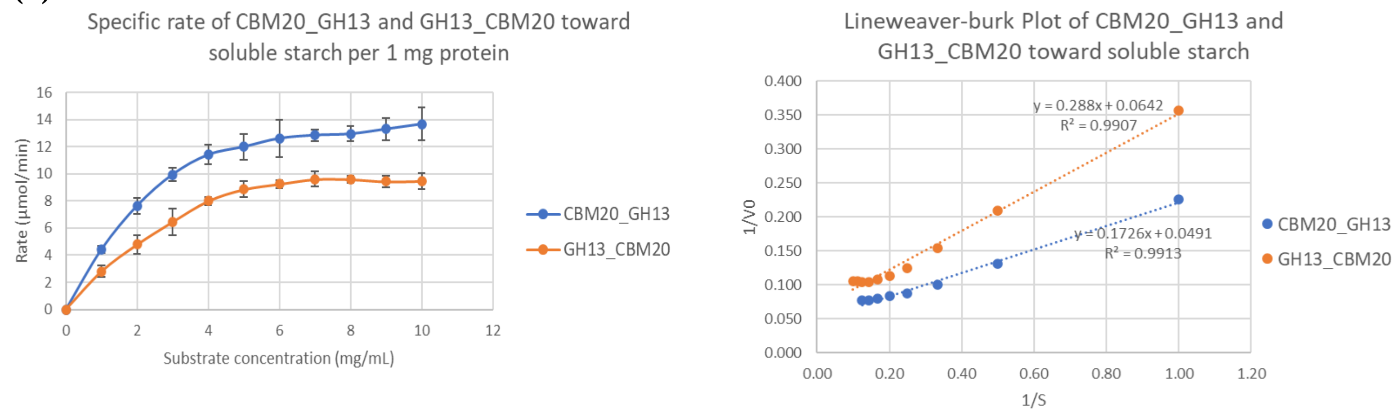

(C)

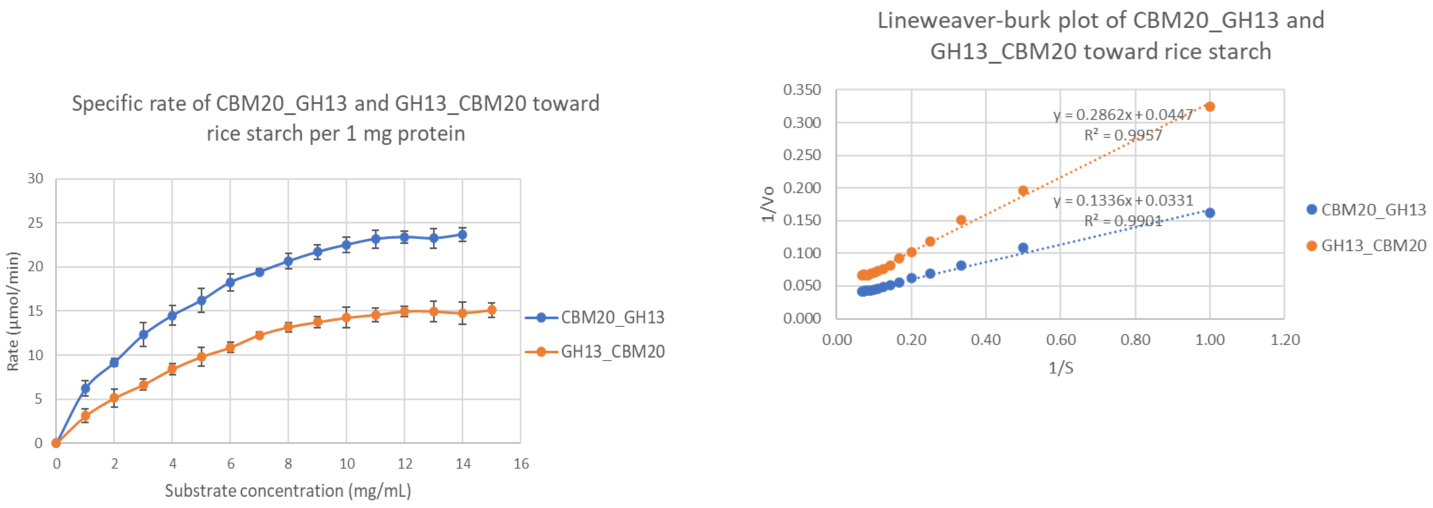

(D)

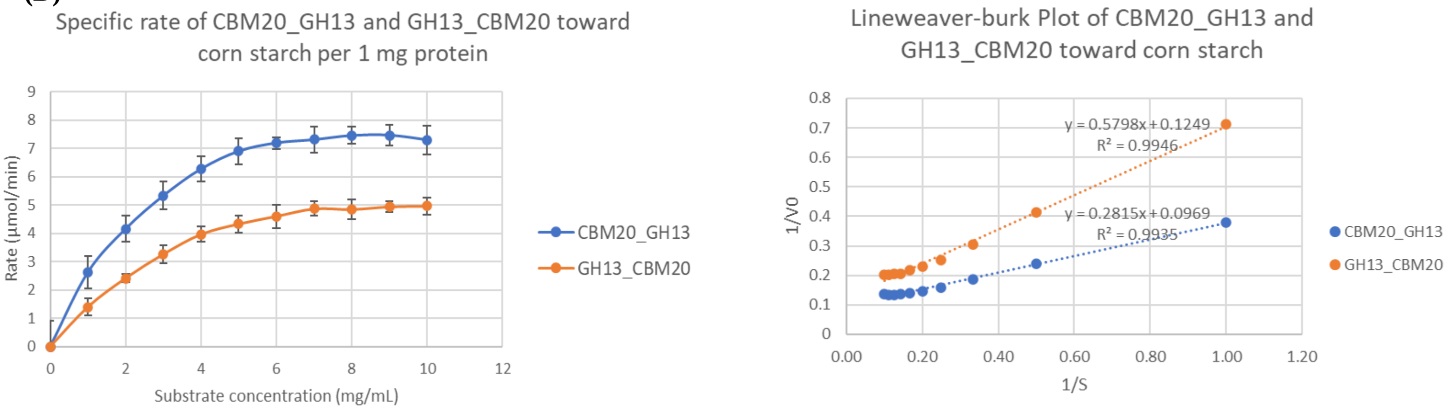
Specific rate of CBM20_GH13 and GH13_CBM20 toward corn starch per 1 mg protein
Rate (µmol/min)
Substrate concentration (mg/mL)
CBM20_GH13
GH13_CBM20
Lineweaver-burk Plot of CBM20_GH13 and GH13_CBM20 toward corn starch
y = 0.5798x + 0.1249
R² = 0.9946
y = 0.2815x + 0.0969
R² = 0.9935
1/V0
1/S
CBM20_GH13
GH13_CBM20

(E)

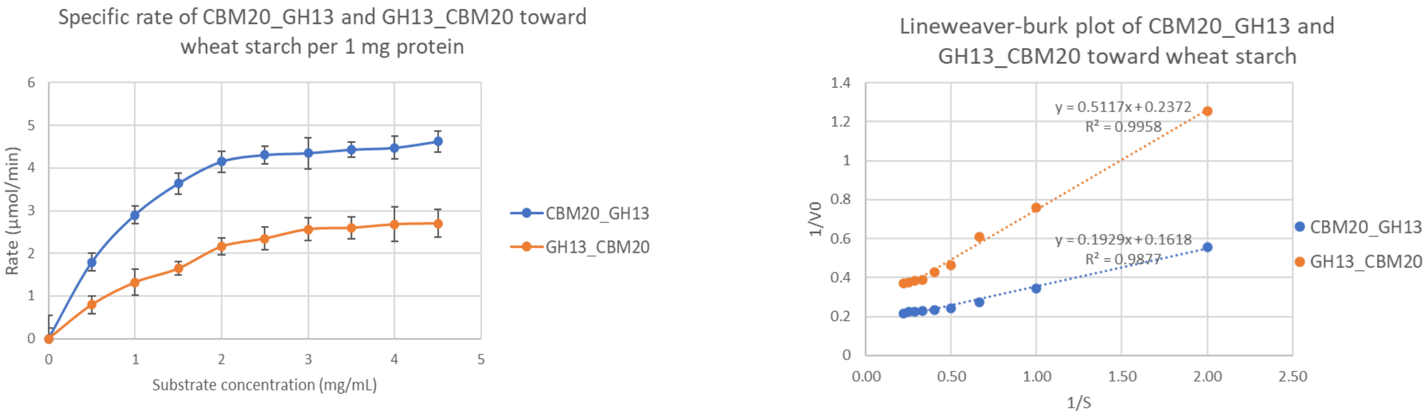
Specific rate of CBM20_GH13 and GH13_CBM20 toward wheat starch per 1 mg protein
Rate (µmol/min)
Substrate concentration (mg/mL)
CBM20_GH13
GH13_CBM20
Lineweaver-burk plot of CBM20_GH13 and GH13_CBM20 toward wheat starch
y = 0.5117x + 0.2372
R² = 0.9958
y = 0.1929x + 0.1618
R² = 0.9877
1/V0
1/S
CBM20_GH13
GH13_CBM20

(F)

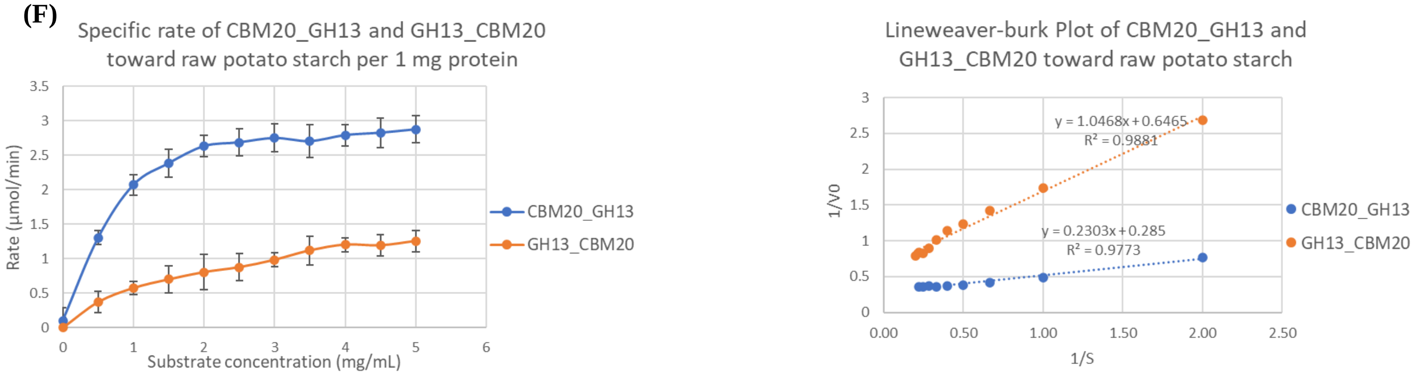
Specific rate of CBM20_GH13 and GH13_CBM20 toward raw potato starch per 1 mg protein
Rate (µmol/min)
Substrate concentration (mg/mL)
CBM20_GH13
GH13_CBM20
Lineweaver-burk Plot of CBM20_GH13 and GH13_CBM20 toward raw potato starch
y = 1.0468x + 0.6465
R² = 0.9881
y = 0.2303x + 0.285
R² = 0.9773
1/V0
1/S
CBM20_GH13
GH13_CBM20
